# Vigilance or avoidance: How do autistic traits and social anxiety modulate attention to the eyes?

**DOI:** 10.3389/fnins.2022.1081769

**Published:** 2023-01-11

**Authors:** Wei Ni, Haoyang Lu, Qiandong Wang, Ci Song, Li Yi

**Affiliations:** ^1^School of Psychological and Cognitive Sciences and Beijing Key Laboratory of Behavior and Mental Health, Peking University, Beijing, China; ^2^Beijing Key Laboratory of Applied Experimental Psychology, National Demonstration Center for Experimental Psychology Education, Faculty of Psychology, Beijing Normal University, Beijing, China; ^3^Education Research Center for Children With ASD, Faculty of Education, Beijing Normal University, Beijing, China; ^4^IDG/McGovern Institute for Brain Research at PKU, Peking University, Beijing, China

**Keywords:** autism spectrum disorder, social anxiety disorder, attention to the eyes, eye avoidance, attention maintenance, eye movement

## Abstract

**Introduction:**

Social anxiety disorder (SAD) and autism spectrum disorder (ASD) are highly overlapping in symptoms and have a high rate of comorbidity, posing challenges in diagnosis and intervention for both disorders. Both disorders are linked to abnormal attention to the eyes, yet how they interactively modulate the attentional process to the eyes remains unclear.

**Methods:**

In this study, we explored how autistic traits and social anxiety in college students separately and together affected different temporal stages of attention to the eyes. Participants were instructed to view virtual faces for 10 s and make an emotional judgment, while their eye movements were recorded.

**Results:**

We found that social anxiety and autistic traits affected different temporal stages of eye-looking. Social anxiety only affected the first fixation duration on the eyes, while autistic traits were associated with eye avoidance at several time points in the later stage. More importantly, we found an interactive effect of autistic traits and social anxiety on the initial attention to the eyes: Among people scoring high on autistic traits, social anxiety was related to an early avoidance of the eyes as well as attention maintenance once fixated on the eyes.

**Discussion:**

Our study suggests the separate and interactive roles of social anxiety and autistic traits in attention to the eyes. It contributes to a deeper understanding of the mechanisms of social attention in both SAD and ASD and highlights the application of psychiatric diagnoses using eye-tracking techniques.

## 1. Introduction

Social anxiety disorder (SAD), characterized by fear and anxiety of social contexts, is one of the most common mental disorders, with a lifetime prevalence of 4% and a 12-month prevalence of 2.4% across countries (Stein et al., 2017). People with SAD worry that their behaviors will be negatively evaluated by others and thus show anxiety and avoidance of various social situations (American Psychiatric Association [APA], 2013). SAD is commonly reported in the typical population (Stein and Stein, 2008) and also among people with special needs, such as people with autism spectrum disorder (ASD), a neurodevelopmental disorder involving social communication impairments and repetitive, restricted behavior and interests (American Psychiatric Association [APA], 2013). Similar to SAD, autistic people also show social avoidance and withdrawn behavior (Kleberg et al., 2017). Moreover, these two disorders have a high rate of comorbidity: Social anxiety affected 12–56% of autistic adults (Rosbrook and Whittingham, 2010; Bejerot et al., 2014; Maddox and White, 2015) and 7–57% of autistic children and adolescence (Leyfer et al., 2006; Kuusikko et al., 2008; White et al., 2015). The overlap of symptoms (e.g., avoidance of social interaction and eye contact) and the high rate of comorbidity between ASD and SAD pose challenges to diagnosis and intervention for both disorders. Hence, an investigation of how these two traits influence social behaviors separately and together becomes necessary. This study focused on the effects of autistic traits and social anxiety on social attention, especially attention to the eyes.

Abnormal attention to the eyes, such as eye avoidance, is commonly reported in people with autistic traits or ASD (e.g., Pelphrey et al., 2002; Chen and Yoon, 2011; Freeth et al., 2013; Yi et al., 2013, 2014), as well as people with SAD (e.g., Schneier et al., 2011; Weeks et al., 2013). The *eye avoidance* hypothesis of autism believes that avoidance of the eyes can cause severe social impairment in autistic people, since the eye region contains the most important social information (Tanaka and Sung, 2016). Autistic people may perceive the eyes as socially threatening, thus avoiding the eyes as an adaptive strategy. Similarly, the *avoidance* hypothesis of SAD proposes that people with SAD use safe actions like avoidance of eye contact to prevent feelings of fear and anxiety in social contexts (Clark and Wells, 1995).

Despite the similar eye avoidance patterns found in autistic people and people with SAD, people with SAD have specific attentional bias to the eyes at the early stages of face processing (e.g., Boll et al., 2016; Keil et al., 2018; Kleberg et al., 2021; Wieser et al., 2009b). The attentional biases in SAD could be explained by *vigilance* theory (Clark and Wells, 1995; Mogg and Bradley, 1998) and *maintenance* or *delayed disengagement* theory (Fox et al., 2002). The *vigilance* theory suggests that people with SAD tend to initially orient their attention toward threatening social information (Bögels and Mansell, 2004). The *maintenance* or *delayed disengagement* theory emphasizes their difficulty in shifting their attention away from social threats (Fox et al., 2002; Amir et al., 2003; Pergamin-Hight et al., 2016; Fernandes et al., 2018). Rapee and Heimberg (1997) suggested that both attentional processes within the same social anxiety model of enhanced attention to the social threats, but the underlying cognitive mechanisms of the two processes differ, which can be differentiated by eye-tracking evidence. Particularly, *vigilance* has been reflected in more first fixations on the eyes (Boll et al., 2016) and shorter latency to the eyes (Keil et al., 2018) in SAD at all ages, while the *maintenance* of attention has been indicated by a longer latency to shift their attention away from threatening faces in adults (Buckner et al., 2010) and from the eye region in children and adolescents with SAD (Kleberg et al., 2021).

Beside the early attentional bias toward the eyes, people with SAD may also experience a later avoidance of the eyes, according to *vigilance-avoidance* hypothesis (Amir et al., 1998; Bögels and Mansell, 2004). Previous studies in SAD have suggested the importance of distinguishing early (*vigilance* or *maintenance*) and late (*avoidance*) attentional patterns regarding the eyes. This dynamic process has been explored using temporal analyses looking at the attentional patterns in different stages (e.g., Wieser et al., 2009a; Keil et al., 2018), but has not been proved by other studies (e.g., Boll et al., 2016; Capriola-Hall et al., 2021). Different from SAD, autistic people show a persistent eye avoidance across time (e.g., Wang et al., 2018).

In summary, although autism and SAD both display atypical attention to the eyes, their gaze patterns and underlying mechanisms could differ. More importantly, autistic traits and social anxiety were highly correlated in both clinical samples and healthy people (Bellini, 2006; Freeth et al., 2013; Bejerot et al., 2014), resulting in their interactive impacts on the gaze pattern to the eyes. On one hand, social anxiety is one of the most common comorbidities of autism (e.g., Maddox and White, 2015), and thus could affect the eye-looking patterns in autistic people. One previous study found that the fear of negative evaluation, a symptom of social anxiety, was related to longer gaze duration to social threats in autism (White et al., 2015). On the other hand, co-occurring autistic traits could contribute to the heterogeneity of attentional patterns to the eyes in people with high social anxiety. Given that autistic traits were related to avoidance of eyes (Chen and Yoon, 2011), socially anxious people with high autistic traits may exhibit more avoidant instead of vigilant or maintained eye-looking patterns. Therefore, exploring the influence of autistic traits and social anxiety on the attention to the eyes is necessary not only for a better understanding of the mechanisms of social attention, but also for the diagnosis and treatment of both disorders.

Autistic traits and social anxiety affect not only clinical samples, but also the general population. Non-autistic people who have familial risks or show more features of autism than the average but do not meet the diagnostic criteria of ASD are defined as the broader autism phenotype (BAP; Wheelwright et al., 2010; Sucksmith et al., 2011). Anxiety disorder, could also be defined as continuums, as suggested by Research Domain Criteria framework (RDoC; Insel et al., 2010). The severity of SAD or ASD symptoms could be reflected by social anxiety and autistic traits, obtained by psychometric measures. These two traits could affect many aspects of social cognitive processes of two disorders in the broader population. More importantly, we could explore how these traits interactively affect cognition by regarding the traits as continuous variables, which would bring insights to the cognitive mechanism of comorbidity.

The current study examined how autistic traits and social anxiety in the general population separately and interactively affected attention to the eyes. We aimed to address the following research questions. First, how do autistic traits and social anxiety separately or interactively modulate attention to the eyes? Here we focus on the interactive effects of these two traits, which have rarely been explored in previous investigations. We hypothesized that social anxiety and autistic traits may interactively influence the attention to the eyes. Second, we separated the stimulus display time into different stages and examined the roles of autistic traits and social anxiety at different times in the stimulus. We examined the roles of autistic traits and social anxiety in attention to the eyes at the very early stages, including the proportion, duration, and latency of the first fixations on the eyes. At the later stages of face processing, we investigated whether autistic traits and social anxiety were associated with more eye avoidance.

## 2. Materials and methods

### 2.1. Participants

Sixty-four healthy college students participated in our experiment. Forty-seven were recruited directly from the campus internal forum at Peking University. To match the number of participants in the high and low autistic traits group, we posted another recruitment advertisement online to screen participants with high AQ scores. Forty-one students completed the online AQ and Social Phobia Inventory (SPIN) questionnaires, out of which 17 scored high in AQ (averaged score >26) and were included in our formal experiment. All the participants were required to be nearsighted below 5.0 diopters, and without colorblindness or color weakness. All participants were required not to be diagnosed with any psychiatric disorders, according to self-report. Four participants were excluded from data analysis for missing data in the questionnaire, eye-tracking calibration problems (See section “2.3 Procedures” for details), or computer crashes during the experiments. The final sample size was 60 (21 males, aged 18–29, mean age = 22.02, SD_*age*_ = 2.45). We separated our participants into high and low autistic traits groups and high and low social anxiety groups. The high autistic traits group consisted of 27 participants, and the high social anxiety group consisted of 37 participants. Twenty-one participants were in both high autistic and high social anxiety groups. More information of our participants was shown in Table 1. The study was approved by the Committee for Protecting Human and Animal Subjects at School of Psychological and Cognitive Sciences at Peking University, China (protocol number: 2020-03-09).

**TABLE 1:** Means (standard deviations) of demographic information and clinical test scores of participants.

	Full sample (*N* = 60)	Group by AQ score		Group by SPIN score	
		High AQ (*N* = 27)	Low AQ (*N* = 33)	High SPIN (*N* = 37)	Low SPIN (*N* = 23)
Age	22.02 (2.45)	21.63 (2.30)	22.33 (2.52)	21.73 (2.37)	22.48 (2.50)
Gender (female/male)	39/21	18/9	21/12	23/14	16/7
**AQ**					
Four-point scale	120.35 (14.28)	131.07 (8.25)	111.58 (12.01)	124.30 (12.84)	114 (14.44)
Binary scale	23.03 (6.52)	28.33 (2.92)	18.70 (5.33)	24.76 (5.81)	20.26 (6.77)
SPIN	24.37 (12.47)	29.85 (13.23)	19.88 (9.91)	31.70 (9.76)	12.57 (4.97)

### 2.2. Materials

#### 2.2.1. Psychometric measures

We used a Chinese version of the Autism-spectrum Quotient (Baron-Cohen et al., 2001) scale to measure participants’ autistic traits. It was a self-report scale with 50 items containing five different autistic dimensions: social skill, attention switching, attention to detail, communication, and imagination (Baron-Cohen et al., 2001). Two scoring approaches were widely used in ASD researches. The first one was the 4-point scoring system (1 = *definitely disagree* to 4 = *definitely agree*), a continuous approach that extended well in the non-clinical samples (Murray et al., 2016). The second one was the dichotomous system (0 = *definitely disagree* or *slightly disagree* and 1 = *slightly agree* or *definitely agree*), which was optimal for diagnosing individuals (Thomas, 2011). We adopted the 4-point version to accurately represent the continuity of autistic traits when conducting linear mixed models (LMMs). In the temporal analysis, we divided our participants into the high and low AQ groups by a cut-off value of 26 in the original AQ dichotomous scale and compared the two groups (Woodbury-Smith et al., 2005). The cut-off value at 26 was found to be a more sensitive score to screen ASD and broader autism phenotype (Woodbury-Smith et al., 2005).

We also used a Chinese version of the Social Phobia Inventory (SPIN) by Connor et al. (2000) to assess social anxiety. It was a self-report scale with 17 items measuring three dimensions: fear, avoidance, and physiological discomfort (Connor et al., 2000). Participants rated from 0 (not at all) to 4 (extremely) according to their situations during the last 3 months (Connor et al., 2000). The higher SPIN score represented, the higher the level of social anxiety. We used the SPIN score as a continuous variable in LMMs, and divided the participants into the high and low SPIN groups according to the cut-off score 19 in temporal analysis (Connor et al., 2000). The correlation between SPIN score and 4-point AQ score was *r* = 0.44 (*p* < 0.001).

#### 2.2.2. Stimulus materials and apparatus

Face stimuli were virtual Asian male faces generated by FaceGen Modeller 3.5 (Singular Inversions, 2010) randomly, including five emotions: angry, fearful, happy, sad, and neutral. Each emotion consisted of eight different face images (400 × 400 pixels). We used the Tobii Pro Spectrum (Tobiitech, Stockholm, Sweden) eye-tracker to collect the eye gaze data with a sample rate of 300 Hz. Stimuli were presented on a 24-inch screen with a resolution of 1,920 × 1,080 pixels.

### 2.3. Procedures

Participants sat in a chair 60 cm away from the screen and were asked to keep their head and body still during the task. Before the experiment, participants needed to perform an eye movement calibration designed by Tobii Pro Spectrum. The calibration was accepted only if the errors of all five points for two eyes were less than 1° visual angle.

**FIGURE 1:**
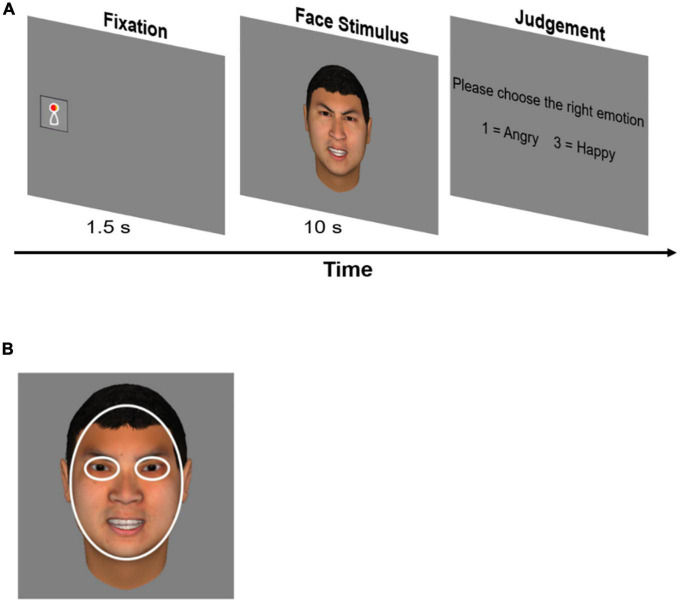
Emotion discrimination task and definition of AOIs. **(A)** Each trial started with an animated fixation that appeared randomly on either left or right side of the screen. Participants needed to fix the fixation for 1.5 s before a central face appeared. This step was designed to measure the latency time and the position of the participant’s first fixations to the central face. The face was presented for 10 s. After that, participants needed to choose the right facial emotion from two options by pressing a key. **(B)** Areas of interest were eyes and face. Fixation data on both eyes of the face were computed together when analyzing data.

Participants were asked to complete an emotion discrimination task, including four blocks with 20 trials in each block. Each trial began with a 3° × 3° animated fixation that appeared randomly on either the left or the right side of the screen to catch participants’ attention. Participants had to fixate on the animation for 1.5 s before a 20° × 20°central face picture appeared. The face picture was presented for 10 s, during which participants were instructed to view the face freely. We used 10 s to explore the temporal dynamics of gaze patterns (van der Geest et al., 2002). After that, participants needed to press the key “1” or “3” to choose the right facial emotion from two options presented on the screen (see Figure 1A). The task was set up to engage the participants.

### 2.4. Data analysis

Eye movement data were analyzed in R (Version 4.0.2). Missing data were filled by linear interpolation with a maximum time gap of 75 ms (Olsen, 2012). We averaged the gaze data from both eyes when calculating fixations. Areas of interest (AOIs) were defined in Figure 1B.

We excluded invalid trials according to the following criteria: (1) trials with more than 30% missing valid eye gaze data (Wang et al., 2020); (2) trials with less than 50% of the time looking at the face out of the whole screen. According to these criteria, a total of 874 (18.2%) invalid trials were excluded. The mean accuracy of emotion judgment was 88.5% (SD = 0.13). ANOVA test revealed no effect of AQ or SPIN on the judgment accuracy (*ps* > 0.05). We excluded incorrect trials in the analyses.

To explore the roles of autistic traits and social anxiety in the attention to the eyes, we focused on three aspects of eye movement data: the overall eye-looking time, the initial attention to the eyes, and the temporal dynamics of eye-looking time. First, for the overall eye-looking time, we calculated the proportional fixation time on the eyes out of the whole face (eyes + rest of the face) during the whole 10 s of the trial. We adopted a linear mixed model with z-transformed AQ, SPIN, and facial emotion as fixed variables and subjects as the random variable to examine the impacts of AQ, SPIN, and facial emotion on the overall eye-looking time. We also included sex of the participants as a covariate in the model since the majority of the participants were female. The LMM was estimated in the “afex” package (Singmann et al., 2021), and *post hoc* comparisons were made in the “emmeans” package (Lenth, 2020). Second, to examine how the two traits separately or interactively affect the initial attention to the eyes, we computed the proportion of first fixations on the eyes when participants looked away from the peripheral fixation to the face, by dividing the trials where the first fixation was on the eyes by the individual number of valid trials with correct answers. Instead of exploring the attentional process of the whole face, we calculated the latency to the eyes (the time from the facial stimulus appearing to the onset of the first fixations on the eyes) and the duration (the eye-looking time of the first fixation on the face) of the first fixations on the eyes because we intended to focus on examining the vigilance, maintenance, and avoidance of the eyes in this study. These theories emphasized on how the individual’s attention was first attracted by the socially threatening stimuli in the environment, which was the eye gaze in our study. Therefore, the first fixation on the eye region was a precondition to our research question. Several studies used the same methods to investigate the early attentional process to the human eye region (e.g., Keil et al., 2018; Capriola-Hall et al., 2021). We then conducted three LMMs to examine the impacts of AQ, SPIN, and facial emotion on the proportion of first fixations on the eyes, first fixation duration, and latency. The descriptive data of overall eye-looking time, proportion of first fixations on the eyes, first fixation duration, and first fixation latency under five emotions were shown in Supplementary Table 1. To better understand how AQ modulate the effect of SPIN, we chose three Z-transformed scores of AQ (-1,0,1) to represent low, medium, and high level of autistic traits in our further simple slope analyses of SPIN. Third, to further explore the temporal dynamics of eye-looking time, we used a moving-average approach (e.g., Dankner et al., 2017; Wang et al., 2018) to calculate the proportional eye-looking time of each 250 ms epoch. We then separated our participants into the high and low autistic trait groups by the cut-off value of 26 in the AQ scale (Woodbury-Smith et al., 2005), or the high and low social anxiety groups by the cut-off value of 19 in SPIN (Connor et al., 2000). We adopted a cluster-based permutation test to compare the differences between different groups of participants (e.g., Wang et al., 2018, 2020).

## 3. Results

### 3.1. Autistic traits or social anxiety do not modulate the overall eye-looking time

We used the proportion of eye-looking time out of face-looking time during the 10 s of the face stimulus as the index of overall eye-looking time. The original full model consisted of the random slopes of emotion within participants and the random intercept of participants. However, the model failed to converge. The ANOVA indicated an insignificant difference between the original and nested models with the random intercept of participants as the only random effect. Therefore, the final model used the main and interaction of autistic traits, social anxiety, and emotion as fixed effects, participants as the random effect, and sex as the covariate. The LMMs below used the same fixed, random, and controlling effects.

**FIGURE 2:**
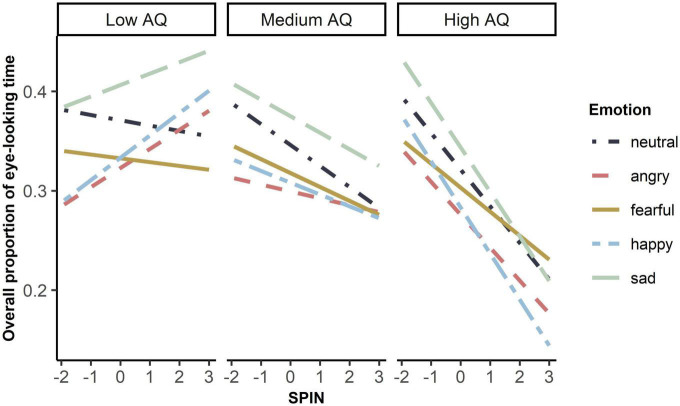
Simple slope plot of interaction between autistic traits, social anxiety, and emotion on overall eye-looking time. Low autistic traits referred to –1 standard deviation below mean AQ (original score: 106). Medium autistic traits referred to mean AQ (original score: 120). High autistic traits referred to 1 standard deviation above mean AQ (original score:135). X-axis was the z-standardized score of SPIN. Y-axis was the original data of overall eye-looking time.

The complete LMM result was shown in Supplementary Table 2. We found no main effect of either autistic traits, *F*(1, 53.82) = 0.99, *p* = 0.33, SPIN, *F*(1, 54.31) = 0.37, *p* = 0.55, or the interaction of autistic traits and social anxiety, *F*(1, 54.26) = 1.32, *p* = 0.26. However, autistic traits, social anxiety, and emotion revealed a significant interaction on the overall eye-looking time, *F*(4, 3384.86) = 3.29, *p* = 0.01. We then compared slopes of social anxiety under five emotions between high (1 SD above the mean AQ), medium (mean AQ), and low levels of autistic traits (-1 SD below the mean AQ), and found no difference under any emotions (*ps* > 0.05, see Supplementary Table 3 for details). Even so, as shown in Figure 2, participants with lower autistic traits tended to look at eyes more as they scored more on social anxiety, while those with higher autistic traits tended to look at eyes less as they scored more on social anxiety. Moreover, sex had an effect on the overall eye-looking time, *F*(1,54.62) = 4.79, *p* = 0.033. Female participants looked longer at the eyes than male participants.

In sum, we did not find direct evidence of impacts of autistic traits and social anxiety on the overall attention to the eyes, independently or interactively. Thus, we focused on their effects on the temporal dynamics of eye-looking patterns in the following analyses.

### 3.2. Autistic traits and social anxiety interactively modulated the first fixations to the eyes

**FIGURE 3:**
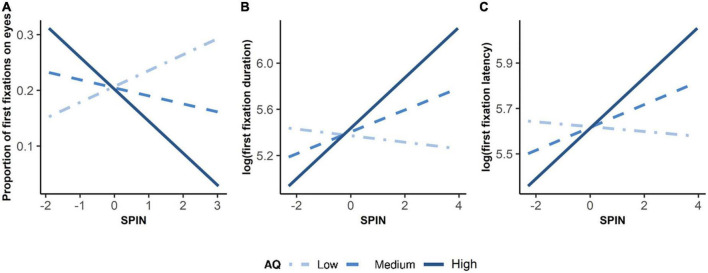
Simple Slope Plots of Interaction between Autistic Traits and Social Anxiety on Initial Attention to the Eyes. Low autistic traits referred to –1 standard deviation below mean AQ. Medium autistic traits referred to mean AQ. High autistic traits referred to 1 standard deviation above mean AQ. X-axis was the z-standardized score of SPIN. Three plots show interactions between autistic traits and social anxiety on **(A)** proportion of first fixations on eyes, **(B)** log-transformed duration of first fixation, and **(C)** log-transformed latency of first fixation.

To investigate how autistic traits and social anxiety impacted the first fixations on the eyes, we adopted three LMMs with dependent variables of the proportion of first fixations on the eyes, the first fixation durations on the eyes, and the latency to the eyes. As shown in Supplementary Table 4, the LMM showed a significant interaction between autistic traits and social anxiety on the proportion of first fixations on the eyes, *F*(1, 50.79) = 4.52, *p* = 0.038. Simple slope analyses revealed that the effect of social anxiety on the proportion of first fixations on the eyes was significant at the high level of autistic traits (1 SD above the mean AQ), *B* = –0.058, *t* = –2.055, β = –0.314, *p* = 0.045. No effect of social anxiety was found at the low (-1 SD below the mean AQ) or medium level of autistic traits (mean AQ), *B* = 0.029, *t* = 0.825, β = 0.156, *p* = 0.413, and *B* = –0.014, *t* = –0.599, β = –0.079, *p* = 0.552, respectively (see Figure 3A). The main effect of social anxiety or autistic traits was not significant, *F*(1, 51.79) = 0.36, *p* = 0.552, and *F*(1, 50.54) = 0.01, *p* = 0.923, respectively. These results indicated that participants with both high autistic and high social anxiety looked less at eyes when they attended to the face, compared to individuals with high autistic traits and low social anxiety.

Then, we selected trials in which participants’ first fixations (29 percent of valid trials) were on the eyes and made log-transformations of the first fixation duration and the latency of first fixation because of non-normality (Shapiro–Wilk normality test, *W* = 0.252, *p* < 0.001, and *W* = 0.657, *p* < 0.001, respectively). We conducted another two LMMs with log-transformed first fixation duration and log-transformed latency as dependent variables of each model.

**FIGURE 4:**
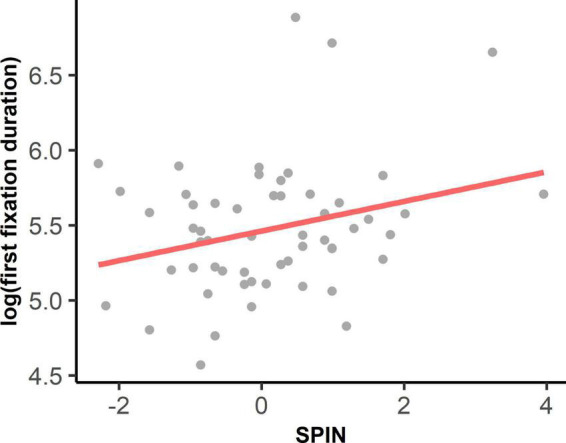
Main effect of social anxiety on log-transformed of first fixation duration at the eyes.

As shown in Supplementary Table 5, the interaction between autistic traits and social anxiety on log-transformed first fixation duration was significant, *F*(1, 56.26) = 7.10, *p* = 0.010. Simple slope analyses showed that social anxiety had a positive effect on the first fixation duration at the eyes at the high level of autistic traits (*B* = 0.219, *t* = 3.434, β = 0.380, *p* = 0.001), as well as the medium level of autistic traits (*B* = 0.095, *t* = 2.007, β = 0.165, *p* = 0.0496). Social anxiety did not show any effect on first fixation duration at the low level of autistic traits (*B* = –0.029, *t* = –0.415, β = –0.050, *p* = 0.680, see Figure 3B). Social anxiety also had a significant main effect, *F*(1, 51.22) = 4.05, *p* = 0.050, indicating that with higher social anxiety, people tended to look at eyes longer on their first fixations (see Figure 4). However, the main effect of autistic traits was not significant, *F*(1, 39.14) = 0.33, *p* = 0.569.

As shown in Supplementary Table 6, social anxiety and autistic traits had a marginal interaction on log-transformed latency, *F*(1, 53.80) = 3.13, *p* = 0.083. Simple slope analyses revealed a positive effect of social anxiety on latency at the high level of autistic traits (*B* = 0.111, *t* = 2.352, β = 0.265, *p* = 0.022), but not at the low (*B* = –0.011, *t* = –0.206, β = –0.025, *p* = 0.837) or the medium levels of autistic traits (*B* = 0.050, *t* = 1.430, β = 0.120, *p* = 0.158, see Figure 3C). The main effect of social anxiety or autistic traits was not significant, *F*(1, 49.49) = 2.05, *p* = 0.158, and *F*(1, 38.11) = 0.01, *p* = 0.936, respectively.

In sum, we found that social anxiety was related to longer first fixation durations on the eyes. In addition, autistic traits and social anxiety had an interactive effect on the first fixations on the eyes. Among people with high autistic traits, social anxiety was related to fewer first fixations on the eyes, longer latency to the eyes, and longer first fixation duration on the eyes. However, among people with low autistic traits, social anxiety was not related to any indices of first fixations on the eyes.

### 3.3. Autistic traits, but not social anxiety, was associated with eye avoidance across time

**FIGURE 5:**
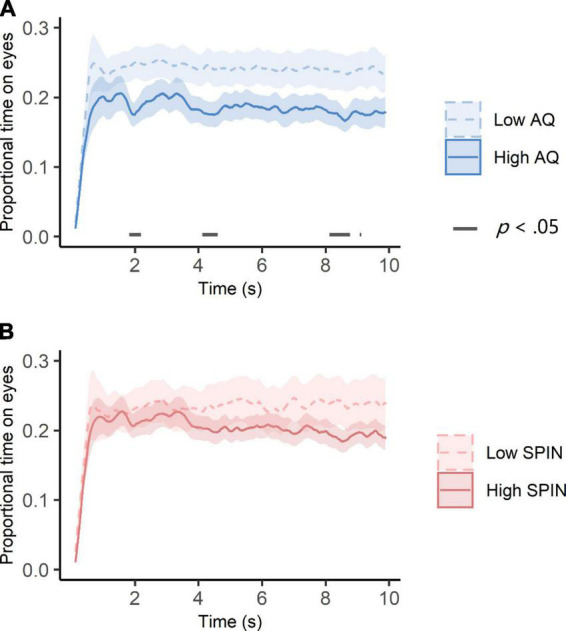
Temporal dynamics of eye-looking time in high and low autistic traits **(A)** and social anxiety **(B)** groups. **(A)** The high autistic traits group consisted of participants whose dichotomous AQ scores were greater than or equal to 26 (*N* = 27). The low autistic traits group consisted of the rest of the participants (*N* = 33). Ribbon areas around lines indicate standard errors. Gray straight lines indicates the cluster of times when group differences of eye-looking time were significant. **(B)** The high social anxiety group consisted of participants whose dichotomous SPIN scores were greater than or equal to 19 (*N* = 37). The low social anxiety group consisted of the rest of the participants (*N* = 23). Ribbon areas around lines indicate standard errors.

Besides the initial attention to the eyes, we also explored how autistic traits and social anxiety impacted eye-looking time at later stages. To this end, we compared temporal dynamics of eye-looking time between the high and low autistic trait groups as well as between the high and low social anxiety groups. The results are shown in Figure 5. People in the low autistic traits group generally looked less at eyes, but the differences were significant at around 2, 4, and 8 s (*p* < 0.05, see Figure 5A). However, there was no difference between high and low social anxiety groups across all time intervals (see Figure 5B).

## 4. Discussion

We aimed to address two research questions in this study to investigate how social anxiety and autistic traits influenced the attention to the eyes. First, we explored how autistic traits and social anxiety separately and interactively influenced the overall attention to the eyes. Our results revealed that the overall eye-looking time was not modulated by either of these two traits. Second, we separated the eye looking by different temporal stages and examined how these two traits affected the attention to the eyes for each stage. We found that these two traits had an interactive impact on the first fixations on the eyes: among people with relatively high autistic traits, social anxiety was related to the avoidance of eyes as well as the early attention maintenance on the eyes. In addition, we found that social anxiety only affected the attention to the eyes on first fixations, while autistic traits affected the avoidance of the eyes significantly at several time points during the 10 s trial.

We found that the effects of social anxiety on attention to the eyes were mainly in the early stage. Specifically, we found that social anxiety could affect first fixation duration on the eyes—the higher the social anxiety, the longer the first fixation duration on the eyes (see Figure 4). Social anxiety did not affect the latency of the first fixation on the eyes or the proportion of the first fixation on the eyes. This finding does not support the *vigilance* hypothesis of SAD, which suggests that the initial vigilance to the eyes is reflected by shorter latency or more first fixations to the eyes in people with SAD (Boll et al., 2016; Keil et al., 2018) and people with relatively high social anxiety (Gutiérrez-García et al., 2018). Instead, our finding of longer first fixation durations on the eyes in high social anxiety indicated an early maintenance of attention to the eyes. The result could be explained by the *maintenance* hypothesis of SAD, which emphasizes the attentional inflexibility of people with SAD when facing social threats (Kleberg et al., 2021). The *maintenance* theory has also been supported by previous studies, which revealed an attentional disengaging problem from socially threatening stimuli in people with SAD (e.g., Fox et al., 2002; Moriya and Tanno, 2011; McGlade et al., 2020) and people with high social anxiety (Wieser et al., 2009a). Although our finding of longer first fixation duration on the eyes in individuals with higher social anxiety was consistent with a previous study in youth with SAD (Capriola-Hall et al., 2021), contradictory findings in Keil et al. (2018) indicated that children with SAD oriented to the eyes faster but their first fixation duration on the eyes was shorter. To further examine the underlying mechanisms of attentional bias in SAD, future studies should use more sophisticated paradigms of disengagement, such as cueing the participants’ initial fixation to the eyes or other socially threatening stimuli, to measure their disengaging time in shifting from the eyes (Boll et al., 2016; Kleberg et al., 2021).

More importantly, we found an interactive effect of social anxiety and autistic traits on the initial attention to the eyes: In people with high autistic traits, social anxiety was related to fewer first fixations and longer latency to the eyes, while in people with low autistic traits, social anxiety did not affect the first fixations on the eyes (as shown in Figures 3A, C). This result indicated that the early *vigilance* to the eyes was not present in people with high social anxiety co-occurring high autistic traits. Instead, autistic traits could enhance the avoidance of the eyes at the early stages of face processing in people with high social anxiety. In addition, social anxiety was related to longer first fixation duration on the eyes in people with high and medium levels of autistic traits, but not in people with low levels of autistic traits (as shown in Figure 3B), indicating the early maintenance of attention to the eyes in people with high social anxiety and relatively high autistic traits. This *maintenance* eye-looking pattern may reflect an early enhanced attention to social stimuli or an impaired disengagement of early attention in people with high social anxiety. Summarizing the results above, our findings suggest that people with high levels of autistic traits and social anxiety tend to avoid looking at the eyes at the early stage, but once they fixated on the eyes, they displayed a tendency to maintain their attention on the eyes. This avoidance-maintenance eye-looking pattern in people with comorbidity of ASD and SAD could interfere the normal interpersonal communication, especially the non-verbal communication, which largely relies on eye contacts and social information processing. Our findings highlight the importance of viewing the effects of the two traits on social attention as interactive rather than separate. Future studies should take into account comorbidities of social anxiety, such as autism, when describing clinical samples, and interpreting the results in future investigations of social attention. Notably, different experimental paradigms should be used to confirm the findings. In Kleberg et al. (2017), participants were presented with the human eye region with three non-social stimuli. After controlling autistic traits, researchers found that people with high social anxiety oriented their attention away from the eyes more quickly. The experimental paradigm and the materials we used might have caused inconsistent findings.

Different from the initial attention to the eyes, later attention to the eyes was only affected by autistic traits, not social anxiety. People with relatively high autistic traits avoided the eyes at several time points at later stage of the trial. This finding supported the *eye avoidance* hypothesis of autism (Tanaka and Sung, 2016), and was consistent with the findings in non-autistic adults with high autistic traits (Chen and Yoon, 2011; Hessels et al., 2018). Inconsistent with the *vigilance-avoidance* account of social anxiety (e.g., Keil et al., 2018), we did not find a later avoidance of the eyes in people with social anxiety. Thus, our findings only support the attentional bias of social anxiety in the early stage, reflecting the subjects’ difficulty in shifting their attention from the eyes (Kleberg et al., 2021). Autistic traits, on the other hand, could be related to an uncomfortable tension elicited by viewing socially threatening stimuli, resulting in a sustained avoidance of the eyes (Tanaka and Sung, 2016). Our study suggests that attention to the eyes in social anxiety among those with autistic traits should be considered as a dynamic process, and different traits could affect different stages of attention to the eyes. However, the underlying neural and physiological mechanisms needs further investigations.

Our study has several implications for the underlying mechanisms and clinical practices regarding SAD and ASD. First, the results contribute to the understanding of heterogeneity of eye-looking patterns in people with SAD. The existing theories have proposed that people with SAD could be vigilant or avoidant of the eyes, or they may be difficult to disengage their attention from the eyes. However, these various gaze patterns could be affected by comorbid situations. Thus, the investigation of social information processing should consider comorbidities, such as co-occurring autism. Second, comorbidity could make the diagnosis of ASD and SAD highly complicated, given their similarities in deficient social information processing. Our study suggests different mechanisms underlying these two traits affecting social attention and may provide an objective tool of eye-tracking techniques for distinguishing the two disorders. Last, our study implies that interventions for autism should also consider co-occurring social anxiety or other disorders for a more individualized and precise intervention approach. Similarly, it is also important for the treatment of social anxiety to consider co-occurring autistic traits.

Our study also had some limitations. First, participants in this study were all college students with different levels of autistic traits and social anxiety. Although people with high level of autistic traits and social anxiety have been found to show similar cognitive processes with clinical samples (e.g., Moriya and Tanno, 2011; Hessels et al., 2018), caution needs to be taken when interpreting the findings and generalizing the conclusions to clinical samples. Second, we recruited a second set of participants with relatively high AQ to match the numbers of participants in the high and low AQ group. Because autistic traits have a moderate correlation with social anxiety (Wainer et al., 2011), the mean scores of SPIN in our participants were therefore higher than the neurotypically sample (Connor et al., 2000). Although the mean score of low SPIN group (i.e., 12) was consistent with previous non-clinical sample data (Connor et al., 2000), it might still be important to note that our sample may not be representative enough. Third, caution should also be taken when generalizing our findings to people in different cultures. Cultural differences could contribute to the differences between our findings and those of previous studies, which were mostly conducted outside of China. Our participants were Chinese college students, who were thought to show more avoidance of eye contacts than Western people in daily communication (Blais et al., 2008; Fu et al., 2012). Therefore, similar studies should be conducted with people from different cultures to confirm our findings (Blais et al., 2008; Fu et al., 2012). Fourth, we had a relatively high rate of invalid trials in the current study which was probably caused by the relatively large trial numbers, simple stimuli and task, and long trial duration. Future studies should use a variety of real human faces with shorter trial duration. We also included only a few trials where the first fixation was on the eyes (21 percent of the original trials) to explore the initial attention to the eyes. Fifth, the measurement of social anxiety in our study (i.e., SPIN) refers to socially anxious symptoms during the last 3 months and could not clearly be considered as a trait measure. Therefore, more research was needed on the effect of trait social anxiety on face processing.

In conclusion, we found that autistic traits and social anxiety could affect different stages of attention to the eyes: social anxiety only affected the first fixations, while autistic traits affected the attention to the eyes across the whole time. More importantly, we found an interactive effect between social anxiety and autistic traits on the attention to the eyes: People with high autistic traits and high social anxiety were initially avoidant of the eyes, but once they looked at the eyes, they maintained their attention on the eyes. Our study suggests an interactive role of different traits on the same cognitive process and emphasizes the importance of considering the temporal dynamic process when investigating the roles of different traits in social attention. Our study also has important implications for the clinical practices of autism and social anxiety.

## Data availability statement

The datasets presented in this study can be found in online repositories. The names of the repository/repositories and accession number(s) can be found below: https://osf.io/eu6rs/.

## Ethics statement

The studies involving human participants were reviewed and approved by Committee for Protecting Human and Animal Subjects at School of Psychological and Cognitive Sciences at Peking University, China. The patients/participants provided their written informed consent to participate in this study.

## Author contributions

WN and CS designed experiments and collected data. WN, HL, and QW conducted the eye-tracking data analysis. WN and HL completed the statistical analysis. WN wrote the manuscript. LY revised the manuscript. All authors contributed to the article and approved the submitted version.
