# Supplementary material for: Vigilance or avoidance: How do autistic traits and social anxiety modulate attention to the eyes?
Source: Front Neurosci. 2023 Jan 11;16:1081769. doi: 10.3389/fnins.2022.1081769 (PMC9876610; doi:10.3389/fnins.2022.1081769)
Supplement: Supplementary file 1 [file Data_Sheet_1.docx]

**Table S1**

*Mean (standard deviation) of the Eye-tracking Parameters under Five Facial Emotions*

|  | Neutral | Angry | Fearful | Happy | Sad |
| --- | --- | --- | --- | --- | --- |
| Overall eye-looking time | 0.35(0.17) | 0.3(0.17) | 0.33(0.17) | 0.3(0.17) | 0.37(0.18) |
| Proportion of first fixation on eyes | 0.19(0.21) | 0.15(0.15) | 0.16(0.16) | 0.2(0.2) | .0.21(0.19) |
| First fixation duration (ms) | 349.75  (733.41) | 262.88  (208.43) | 344.17  (334.68) | 283.94  (187.73) | 244.3  (95.93) |
| First fixation latency | 357  (276.06) | 312.48  (163.31) | 301.27  (137.68) | 280.44  (86.71) | 312.81  (160.82) |

**Table S2**

*Linear Mixed Model of Overall Eye-looking Time*

| Fixed Effects | | | | | | | |
| --- | --- | --- | --- | --- | --- | --- | --- |
|  | Estimate | *SE* | 95% CI | | *t* | | *p* |
| (Intercept) | 3.29e-01 | 2.28e-02 | [0.28 0.37] | | 14.47 | | <.001*** |
| AQ | -2.40e-02 | 2.41e-02 | [-0.07 0.02] | | -0.99 | | .325 |
| SPIN | -1.41e-02 | 2.32e-02 | [-0.06 0.03] | | -0.62 | | .545 |
| Emotion: angry | 1.68e-02 | 5.39e-03 | [0.01 0.03] | | 3.11 | | .002** |
| Emotion: fearful | -2.99e-02 | 5.36e-03 | [-0.04 -0.02] | | -5.58 | | <.001*** |
| Emotion: happy | -1.14e-02 | 5.48e-03 | [-0.02 0] | | -2.09 | | .037* |
| Emotion: sad | -2.12e-02 | 5.39e-03 | [-0.03 -0.01] | | -3.93 | | <.001*** |
| Sex: male | -4.67e-02 | 2.13e-02 | [-.0.9 0] | | -2.19 | | .033* |
| AQ×SPIN | -2.29e-02 | 2.00e-02 | [-0.06 0.02] | | -1.15 | | .256 |
| AQ×Emotion: angry | -9.62e-04 | 5.80e-03 | [-0.01 0.01] | | -0.17 | | .869 |
| AQ×Emotion: fearful | 3.56e-04 | 5.70e-03 | [-0.01 0.01] | | 0.06 | | .950 |
| AQ×Emotion: happy | 9.22e-03 | 5.78e-03 | [0 0.02] | | 1.59 | | .111 |
| AQ×Emotion: sad | -1.16e-03 | 5.57e-03 | [-0.01 0.01] | | -0.21 | | .835 |
| SPIN×Emotion: angry | -6.98e-03 | 5.63e-03 | [-0.02 0] | | -1.24 | | .216 |
| SPIN×Emotion: fearful | 7.18e-03 | 5.54e-03 | [0 0.02] | | 1.30 | | .195 |
| SPIN×Emotion: happy | 1.33e-04 | 5.63e-03 | [-0.01 0.01] | | 0.02 | | .981 |
| SPIN×Emotion: sad | 2.22e-03 | 5.70e-03 | [-0.01 0.01] | | 0.39 | | .697 |
| AQ×SPIN×Emotion: angry | 7.19e-03 | 4.82e-03 | [0 0.02] | | 1.49 | | .135 |
| AQ×SPIN×Emotion: fearful | -3.26e-03 | 4.72e-03 | [-0.01 0.01] | | -0.69 | | .490 |
| AQ×SPIN×Emotion: happy | 1.28e-02 | 4.75e-03 | [0 0.02] | | 2.69 | | .007** |
| AQ×SPIN×Emotion: sad | -1.15e-02 | 4.90e-03 | [-0.02 0] | | -2.34 | | .019* |
| Random Effects | | | | | | | |
|  | | | | Variance | | *S.D.* | |
| Participant (Intercept) | | | | 0.02 | | 0.16 | |
| Model Fit | | | | | | | |
|  | | | | Marginal | | Conditional | |
| *R^2^* | | | | .09 | | .56 | |
| *Note*. *p*-values for fixed effects have been calculated using Satterthwaite's method.  Confidence Intervals have been calculated using the Wald method.  Model equation: Overall eye looking time ~ AQ × SPIN × Emotion + Sex + (1 \| Participant) | | | | | | | |

**Table S3**

*Simple Slope Analysis of Overall Eye-looking Time on SPIN for Three Levels of AQ and Five Emotions*

|  | *B* | *SE* | 95% CI | *t* | *p* |
| --- | --- | --- | --- | --- | --- |
| AQ = -1 *SD* | | | | | |
| Neutral | -0.005 | 0.034 | [-0.074 0.064] | -0.16 | .88 |
| Angry | 0.019 | 0.034 | [-0.050 0.088] | 0.56 | .58 |
| Fearful | -0.004 | 0.035 | [-0.073 0.065] | -0.11 | .91 |
| Happy | 0.023 | 0.035 | [-0.047 0.092] | 0.65 | .52 |
| Sad | 0.011 | 0.035 | [-0.058 0.081] | 0.33 | .74 |
| AQ = M | | | | | |
| Neutral | -0.021 | 0.024 | [-0.069 0.027] | -0.89 | .38 |
| Angry | -0.007 | 0.024 | [-0.055 0.041] | -0.29 | .77 |
| Fearful | -0.014 | 0.024 | [-0.062 0.034] | -0.59 | .56 |
| Happy | -0.011 | 0.024 | [-0.060 0.036] | -0.50 | .62 |
| Sad | -0.017 | 0.024 | [-0.065 0.031] | -0.69 | .49 |
| AQ = 1 SD | | | | | |
| Neutral | -0.037 | 0.028 | [-0.093 0.019] | -1.31 | .19 |
| Angry | -0.033 | 0.028 | [-0.089 0.022] | -1.18 | .24 |
| Fearful | -0.024 | 0.028 | [-0.080 0.032] | -0.86 | .39 |
| Happy | -0.046 | 0.028 | [-0.102 0.010] | -1.65 | .10 |
| Sad | -0.045 | 0.028 | [-0.102 0.012] | -1.58 | .12 |

**Table S4**

*Linear Mixed Model of Proportion of First Fixation on the Eyes*

| Fixed Effects | | | | | | | | | |
| --- | --- | --- | --- | --- | --- | --- | --- | --- | --- |
|  | Estimate | *SE* | 95% CI | | | *t* | | | *p* |
| (Intercept) | 2.05e-01 | 2.37e-02 | [0.16 0.25] | | | 8.63 | | | <.001*** |
| AQ | -2.39e-03 | 2.45e-02 | [-0.05 0.05] | | | -0.10 | | | .923 |
| SPIN | -1.44e-02 | 2.41e-02 | [-0.06 0.03] | | | -0.60 | | | .552 |
| Emotion: angry | -2.42e-02 | 1.43e-02 | [-0.05 0] | | | -1.70 | | | .092 |
| Emotion: fearful | -1.72e-02 | 1.43e-02 | [-0.05 0.01] | | | -1.21 | | | .230 |
| Emotion: happy | 1.40e-02 | 1.42e-02 | [-0.01 0.04] | | | 0.99 | | | .326 |
| Emotion: sad | 2.67e-03 | 1.42e-02 | [-0.03 0.03] | | | 0.19 | | | .851 |
| Sex: male | -9.19e-03 | 2.22e-02 | [-0.05 0.03] | | | -0.41 | | | .681 |
| AQ×SPIN | -4.30e-02 | 2.02e-02 | [-0.08 0] | | | -2.13 | | | .038* |
| AQ×Emotion: angry | 2.16e-02 | 1.52e-02 | [-0.01 0.05] | | | 1.43 | | | .155 |
| AQ×Emotion: fearful | -2.00e-02 | 1.52e-02 | [-0.05 0.01] | | | -1.32 | | | .189 |
| AQ×Emotion: happy | -1.94e-02 | 1.51e-02 | [-0.05 0.01] | | | -1.28 | | | .201 |
| AQ×Emotion: sad | 8.10e-03 | 1.51e-02 | [-0.02 0.04] | | | 0.54 | | | .593 |
| SPIN×Emotion: angry | -9.08e-03 | 1.50e-02 | [-0.04 0.02] | | | -0.61 | | | .544 |
| SPIN×Emotion: fearful | 1.63e-02 | 1.50e-02 | [-0.01 0.05] | | | 1.09 | | | .278 |
| SPIN×Emotion: happy | 3.43e-04 | 1.52e-02 | [-0.03 0.03] | | | 0.02 | | | .982 |
| SPIN×Emotion: sad | -2.45e-03 | 1.49e-02 | [-0.03 0.03] | | | -0.16 | | | .870 |
| AQ×SPIN×Emotion: angry | 2.49e-03 | 1.25e-02 | [-0.02 0.03] | | | 0.20 | | | .843 |
| AQ×SPIN×Emotion: fearful | -3.88e-03 | 1.25e-02 | [-0.03 0.02] | | | -0.31 | | | .757 |
| AQ×SPIN×Emotion: happy | -7.08e-03 | 1.27e-02 | [-0.03 0.02] | | | -0.56 | | | .579 |
| AQ×SPIN×Emotion: sad | 6.02e-04 | 1.25e-02 | [-0.02 0.03] | | | 0.05 | | | .962 |
| Random Effects | | | | | | | | | |
|  | | | | Variance | | | *S.D.* | | |
| Participant (Intercept) | | | | | 0.02 | | | 0.16 | |
| Model Fit | | | | | | | | | |
|  | | | | Marginal | | | Conditional | | |
| *R^2^* | | | | .08 | | | .68 | | |
| *Note*. *p*-values for fixed effects have been calculated using Satterthwaite's method.  Confidence Intervals have been calculated using the Wald method.  Model equation: Proportion of first fixation on the eyes ~ AQ × SPIN × Emotion + Sex + (1 \| Participant) | | | | | | | | | |

**Table S5**

*Linear Mixed Model of Log-transformed First Fixation Duration on the Eyes*

| Fixed Effects | | | | | | | | | | | |
| --- | --- | --- | --- | --- | --- | --- | --- | --- | --- | --- | --- |
|  | Estimate | *SE* | 95% CI | | | | *t* | | | | *p* |
| (Intercept) | 5.4045 | 0.0551 | [5.3 5.5] | | | | 98.13 | | | | <.001*** |
| AQ | 0.0320 | 0.0558 | [-0.08 0.14] | | | | 0.57 | | | | .569 |
| SPIN | 0.0950 | 0.0472 | [0 0.19] | | | | 2.01 | | | | .050* |
| Emotion: angry | -0.0445 | 0.0413 | [-0.13 0.04] | | | | -1.08 | | | | .281 |
| Emotion: fearful | 0.1077 | 0.0420 | [0.03 0.19] | | | | 2.57 | | | | .011* |
| Emotion: happy | 0.0164 | 0.0396 | [-0.06 0.09] | | | | 0.41 | | | | .680 |
| Emotion: sad | -0.0687 | 0.0402 | [-0.15 0.01] | | | | -1.71 | | | | .088 |
| Sex: male | 0.0160 | 0.0547 | [-0.09 0.12] | | | | 0.29 | | | | .771 |
| AQ×SPIN | 0.1236 | 0.0464 | [0.03 0.21] | | | | 2.67 | | | | .010* |
| AQ×Emotion: angry | 0.0045 | 0.0425 | [-0.08 0.09] | | | | 0.11 | | | | .915 |
| AQ×Emotion: fearful | -0.0519 | 0.0441 | [-0.14 0.03] | | | | -1.18 | | | | .239 |
| AQ×Emotion: happy | 0.0259 | 0.0404 | [-0.05 0.11] | | | | 0.64 | | | | .522 |
| AQ×Emotion: sad | -0.0535 | 0.0387 | [-0.13 0.02] | | | | -1.38 | | | | .167 |
| SPIN×Emotion: angry | -0.0660 | 0.0425 | [-0.15 0.02] | | | | -1.55 | | | | .121 |
| SPIN×Emotion: fearful | 0.0891 | 0.0432 | [0 0.17] | | | | 2.06 | | | | .040* |
| SPIN×Emotion: happy | -0.0050 | 0.0418 | [-0.09 0.08] | | | | -0.12 | | | | .904 |
| SPIN×Emotion: sad | -0.0383 | 0.0421 | [-0.12 0.04] | | | | -0.91 | | | | .363 |
| AQ×SPIN×Emotion: angry | -0.0169 | 0.0504 | [-0.12 0.08] | | | | -0.34 | | | | .737 |
| AQ×SPIN×Emotion: fearful | -0.0268 | 0.0440 | [-0.11 0.06] | | | | -0.61 | | | | .543 |
| AQ×SPIN×Emotion: happy | 0.0278 | 0.0455 | [-0.06 0.12] | | | | 0.61 | | | | .542 |
| AQ×SPIN×Emotion: sad | 0.0587 | 0.0470 | [-0.03 0.15] | | | | 1.25 | | | | .212 |
| Random Effects | | | | | | | | | | | |
|  | | | | Variance | | | | *S.D.* | | | |
| Participant (Intercept) | | | | | | 0.10 | | | | 0.32 | |
| Model Fit | | | | | | | | | | | |
|  | | | | | Marginal | | | | Conditional | | |
| *R^2^* | | | | | .09 | | | | .36 | | |
| *Note*. *p*-values for fixed effects have been calculated using Satterthwaite's method.  Confidence Intervals have been calculated using the Wald method.  Model equation: First fixation duration on the eyes ~ AQ × SPIN × Emotion + Sex + (1 \| Participant) | | | | | | | | | | | |

**Table S6**

*Linear Mixed Model of Log-transformed First Fixation Latency on the Eyes*

| Fixed Effects | | | | | |
| --- | --- | --- | --- | --- | --- |
|  | Estimate | *SE* | 95% CI | *t* | *p* |
| (Intercept) | 5.6163 | 0.0411 | [5.54 5.70] | 136.66 | <.001*** |
| AQ | -0.0034 | 0.0417 | [-0.09 0.08] | -0.08 | .936 |
| SPIN | 0.0503 | 0.0351 | [-0.02 0.12] | 1.43 | .158 |
| Emotion: angry | 0.0261 | 0.0296 | [-0.03 0.08] | 0.88 | .378 |
| Emotion: fearful | -0.0192 | 0.0301 | [-0.08 0.04] | -0.64 | .525 |
| Emotion: happy | -0.0465 | 0.0284 | [-0.1 0.01] | -1.64 | .102 |
| Emotion: sad | 0.0149 | 0.0288 | [-0.04 0.07] | 0.52 | .606 |
| Sex: male | 0.0067 | 0.0407 | [-0.07 0.09] | 0.17 | .870 |
| AQ×SPIN | 0.0609 | 0.0344 | [-0.01 0.13] | 1.77 | .083 |
| AQ×Emotion: angry | 0.0078 | 0.0305 | [-0.05 0.07] | 0.26 | .799 |
| AQ×Emotion: fearful | 0.0139 | 0.0316 | [-0.05 0.08] | 0.44 | .661 |
| AQ×Emotion: happy | -0.0348 | 0.0290 | [-0.09 0.02] | -1.20 | .230 |
| AQ×Emotion: sad | -0.0139 | 0.0278 | [-0.07 0.04] | -0.50 | .617 |
| SPIN×Emotion: angry | -0.0329 | 0.0305 | [-0.09 0.03] | -1.08 | .280 |
| SPIN×Emotion: fearful | 0.0026 | 0.0310 | [-0.06 0.06] | 0.09 | .932 |
| SPIN×Emotion: happy | 0.0375 | 0.0300 | [-0.02 0.1] | 1.25 | .212 |
| SPIN×Emotion: sad | 0.0017 | 0.0302 | [-0.06 0.06] | 0.06 | .955 |
| AQ×SPIN×Emotion: angry | 0.0063 | 0.0362 | [-0.06 0.08] | 0.18 | .861 |
| AQ×SPIN×Emotion: fearful | 0.0178 | 0.0316 | [-0.04 0.08] | 0.57 | .572 |
| AQ×SPIN×Emotion: happy | -0.0536 | 0.0327 | [-0.12 0.01] | -1.64 | .101 |
| AQ×SPIN×Emotion: sad | 0.0242 | 0.0338 | [-0.04 0.09] | 0.72 | .474 |
| Random Effects | | | | | |
|  | | | Variance | *S.D.* | |
| Participant (Intercept) | | | 0.06 | 0.24 | |
| Model Fit | | | | | |
|  | | | Marginal | Conditional | |
| *R^2^* | | | .04 | .35 | |
| *Note*. *p*-values for fixed effects have been calculated using Satterthwaite's method.  Confidence Intervals have been calculated using the Wald method.  Model equation: First fixation latency on the eyes ~ AQ × SPIN × Emotion + Sex + (1 \| Participant) | | | | | |
